# Functional analysis of cancer-associated EGFR mutants using a cellular assay with YFP-tagged EGFR intracellular domain

**DOI:** 10.1186/1476-4598-6-56

**Published:** 2007-09-18

**Authors:** Matheus M de Gunst, Marielle I Gallegos-Ruiz, Giuseppe Giaccone, Jose Antonio Rodriguez

**Affiliations:** 1 Department of Medical Oncology, VU University Medical Center, Amsterdam, The Netherlands; 2 Medical Oncology Branch, CCR, National Cancer Institute, NIH, Bethesda, MD 20892-1906, USA; 3 Department of Genetics, Physical Anthropology and Animal Physiology, University of the Basque Country, 48940 Leioa, Spain

## Abstract

**Background:**

The presence of EGFR kinase domain mutations in a subset of NSCLC patients correlates with the response to treatment with the EGFR tyrosine kinase inhibitors gefitinib and erlotinib. Although most EGFR mutations detected are short deletions in exon 19 or the L858R point mutation in exon 21, more than 75 different EGFR kinase domain residues have been reported to be altered in NSCLC patients. The phenotypical consequences of different EGFR mutations may vary dramatically, but the majority of uncommon EGFR mutations have never been functionally evaluated.

**Results:**

We demonstrate that the relative kinase activity and erlotinib sensitivity of different EGFR mutants can be readily evaluated using transfection of an YFP-tagged fragment of the EGFR intracellular domain (YFP-EGFR-ICD), followed by immunofluorescence microscopy analysis. Using this assay, we show that the exon 20 insertions Ins770SVD and Ins774HV confer increased kinase activity, but no erlotinib sensitivity. We also show that, in contrast to the common L858R mutation, the uncommon exon 21 point mutations P848L and A859T appear to behave like functionally silent polymorphisms.

**Conclusion:**

The ability to rapidly obtain functional information on EGFR variants of unknown relevance using the YFP-EGFR-ICD assay might prove important in the future for the management of NSCLC patients bearing uncommon EGFR mutations. In addition, our assay may be used to determine the response of resistant EGFR mutants to novel second-generation TKIs.

## Background

Approximately 80% of lung cancers, the most frequently diagnosed type of human tumor, are classified as non-small cell lung cancer (NSCLC). Novel therapeutic agents for the treatment of NSCLC patients are currently under intense experimental and clinical investigation, with the goal of increasing their antitumor effect while reducing general toxicity. These agents specifically target cellular pathways necessary for the survival of cancer cells. The epidermal growth factor receptor (EGFR) is a receptor tyrosine kinase (TK) whose activation initiates signal transduction through critical cellular pathways, such as those mediated by Akt and ERK, and thus plays an important role in controlling cell homeostasis [1]. EGFR is overexpressed or aberrantly activated in different types of human tumors, contributing to the malignant phenotype of cancer cells, and targeted inactivation of EGFR is being intensively explored as a cancer therapeutic approach [2]. As a result of these investigations, several small-molecule EGFR tyrosine-kinase inhibitors (TKIs), such as gefitinib and erlotinib, have been developed and are currently available in the clinic. In large clinical studies of gefitinib and erlotinib, it became apparent that a minor subset of NSCLC patients is extremely sensitive to treatment with EGFR-TKIs [reviewed in [3]]. Subsequently, the analysis of EGFR gene sequence revealed the presence of somatic mutations in the kinase domain of the receptor in most responding patients [4-6]. The association between the presence of EGFR mutations and response to TKIs has been confirmed through the analysis of thousands of NSCLC tumor samples worldwide. These results raise the possibility that EGFR mutational analysis may be implemented for the management of NSCLC patients [7].

Approximately 80% of the EGFR mutations detected are short deletions in exon 19 affecting the amino acid sequence ELREA (Del746-750), or a point mutation in exon 21 resulting in the amino acid change L858R. However, the data accumulated in the past three years have uncovered the large allelic heterogeneity that characterizes EGFR kinase mutations. Thus, a survey of the COSMIC mutation database [8] shows that more than 75 different EGFR kinase domain residues have been reported to be altered in NSCLC patients.

The functional characteristics of the two most common types of EGFR alterations, the exon 19 deletions and the L858R point mutation, have been studied in detail using biochemical assays, cell-based systems and mouse models [4-6], [9-14]. Additionally, a limited number of less common mutant alleles of EGFR have been tested using transfection-based approaches [15-22]. Nevertheless, the biological effect of most uncommon EGFR alterations has never been evaluated. The phenotypical effect of the particular alteration detected in tumor cells may largely account for the response of the patient to treatment. In this regard, certain mutations, such as the T790M amino acid change, have been shown to confer resistance to gefitinib and erlotinib [reviewed in [7]]. Second-generation TKIs, which bind covalently to EGFR and may be active against these resistant mutants, are currently being developed.

To allow for a more rapid characterization of untested EGFR mutants, and to facilitate the testing of novel potential anti-EGFR agents, we aimed here to establish a simple cellular assay to evaluate the effect of EGFR mutations and the response of different EGFR variants to erlotinib. To this end, we used site-directed mutagenesis to introduce cancer-associated mutations into an YFP-tagged fragment of EGFR intracellular domain (YFP-EGFR-ICD). These chimerical proteins were transiently expressed in human cells, and the effect of their expression was assessed on a single-cell basis using immunofluorescence with phosphorylation-specific antibodies.

We demonstrate here that the YFP-EGFR-ICD-based assay can be used to evaluate the relative kinase activity and erlotinib sensitivity of EGFR mutants, and we use this approach to test several uncommon EGFR mutations.

## Results

### Increased autophosphorylation of YFP-tagged EGFR intracellular domain the common EGFR Del746 mutation

**Figure 1:**
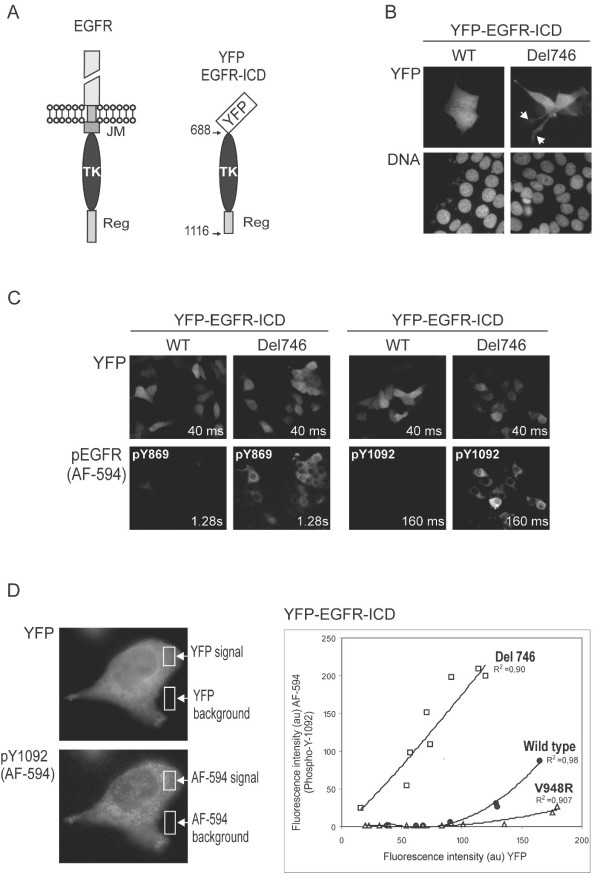
**Common NSCLC-associated EGFR mutations lead to increased autophosphorylation of an YFP-tagged EGFR intracellular domain**. *A*. Schematic representation of full-length EGFR and YFP-EGFR-ICD. The amino acid numbering includes the 24 residues of the signal peptide. YFP-EGFR-ICD contains the tyrosine kinase (TK) domain and part of the regulatory region (Reg), but lacks the extracellular and juxtamembrane (JM) domains. *B*. Expression of YFP-EGFR-ICD Del746 induces morphological changes in MCF-7 cells. Unlike cells transfected with wild type (WT) YFP-EGFR-ICD, MCF-7 cells expressing YFP-EGFR-ICD Del746 frequently show long lamellipodial protrusions (arrowheads). *C *Using immunofluorescence, increased autophosphorylation of YFP-EGFR-ICD Del746 at tyrosine residues Y869 (left set of panels) and Y1092 (right set of panels) can be detected. Phosphorylation is virtually undetectable in cells expressing YFP-EGFR-ICD WT. Images were taken using 160 × magnification and the exposure time indicated inside the panels. The fluorescent signal was consistently brighter using the anti-pY1092 antibody (note the shorter exposure time used). *D*. Semi-quantitative comparison of YFP-EGFR-ICD autophosphorylation level using computer-assisted image analysis. Images of several transfected cells (400 × magnification) were taken using 40 ms (YFP) or 160 ms (AF-594) exposure times. The fluorescence intensity in the green and the red channels was measured within a cytoplasmic area (YFP signal and AF-594 signal), and within an area outside the cells (background). In the graph, the intensity of the YFP and AF-594 fluorophores for each cell was plotted against each other using Excel, and the best-fitting trend lines (highest R^2^) were added. At similar expression levels (YFP intensity), the level of pY1092 is higher for YFP-EGFR-ICD bearing the Del746 mutation (white squares) than for the wild type protein (circles). The V948R mutation (open triangles) virtually abrogated autophosphorylation. The experiment was repeated twice with similar results. Graph shows the data from one experiment. au: arbitrary units.

We generated an YFP-tagged fragment of EGFR (Figure 1A) encompassing residues 688–1116 (the numbering system includes the 24 amino acid signal peptide of EGFR). This fragment, termed YFP-EGFR-ICD, contains the TK domain, as well as sequences from the adjacent regulatory motif, but lacks the extracellular and the juxtamembrane domains of the receptor. The Del746-750 mutation (hereafter called Del746) was next introduced into YFP-EGFR-ICD using site-directed mutagenesis. This mutant, representative of the common and well-characterized exon 19 deletions, was used as a positive control in the initial experiments to test the suitability of our system.

MCF-7 breast cancer cells were transfected with plasmids encoding YFP-EGFR-ICD wt or YFP-EGFR-ICD Del746, and examined using fluorescence microscopy. Approximately 30% of the cells expressing the mutant EGFR ICD showed long lamellipodial protrusions, which were not observed in cells expressing the wt fragment (Figure 1B). Cells were fixed 24 hours after transfection and immunostained using specific primary antibodies to detect phosphorylation of EGFR tyrosine residues Y869 and Y1092. Using secondary antibodies conjugated to the red fluorophore Alexa Fluor-594 (AF-594) and YFP positivity as a marker of transfection, we were able to examine YFP-EGFR-ICD phosphorylation in a single-cell basis (Figure 1C). Non-transfected MCF-7 cells did not contain detectable levels of phosphorylated EGFR. YFP-EGFR-ICD wt-transfected cells showed no or barely detectable pY869 or pY1092. In contrast, cells expressing comparable levels of the mutant protein (as indicated by the intensity of the YFP signal) showed a robust immunostaining signal for both residues. The anti-pY1092 antibody provided the clearest result, and was therefore used in subsequent analyses. Similar experiments were carried out with a shorter EGFR fragment containing only the TK domain (amino acids 688–982), but no autophosphorylation (pY869) was detected (data not shown).

We used computer-assisted image analysis to measure the intensity of the YFP and AF-594 signals in the cytoplasm of several individual cells (Figure 1D, left images). In addition to wt and Del746, an YFP-EGFR-ICD protein bearing the experimental V948R mutation was tested. This amino acid change has been shown to maintain EGFR kinase domain in an inactive conformation [23]. In line with previous data, autophosphorylation was dramatically increased by the Del746 mutation (Figure 1D, graph). It should be pointed out that cells expressing very high levels of the wt ICD showed weak but detectable Y1092 phosphorylation. The V948R change, as expected, virtually abrogated ICD autophosphorylation even at the highest levels of expression.

Altogether, these results demonstrate that the effect of NSCLC-related EGFR mutations on basal autophosphorylation can be rapidly evaluated on a single cell basis using transient transfection of YFP-EGFR-ICD and immunofluorescence.

### Activation of EGFR downstream signaling pathways in cells expressing mutant YFP-EGFR-ICD Del746

Signal transduction downstream of EGFR and, ultimately, the cellular response to EGFR activation, relies on the integrated activity of several intracellular signaling pathways, such as those mediated by Akt or ERK. Phosphorylation of ERK and Akt at specific residues, which constitutes a key activating event in these pathways, is widely used as a marker of active EGFR downstream signaling.

**Figure 2:**
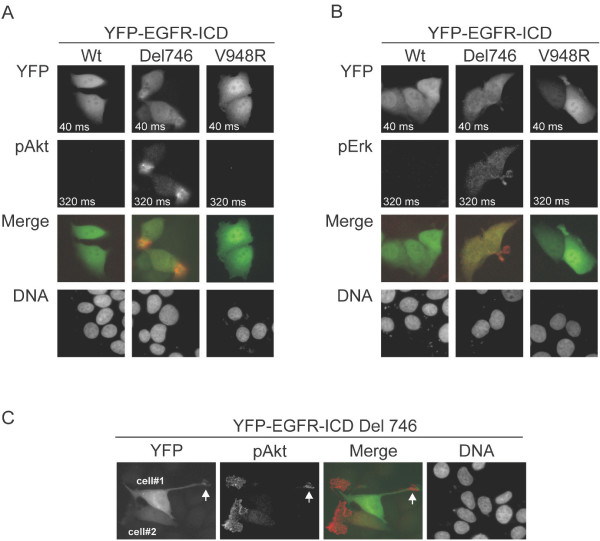
**Activation of Akt and Erk pathways in cells expressing mutant YFP-EGFR-ICD Del746**. A. Panels show representative images (400X) of MCF-7 cells expressing YFP-EGFR-ICD wild type, Del746 or V948R, analyzed by immunofluorescence to detect phosphorylated Akt (pAkt). Phosphorylation of endogenous Akt was only detected in cells expressing the Del746-bearing protein. B. A similar analysis was carried out to detect phosphorylated ERK (pERK). Only cells expressing YFP-EGFR-ICD Del746 contained detectable levels of endogenous pERK. Exposure time is indicated inside the panels. DNA was counterstained with Hoechst. C. Images (400X) illustrate two morphological characteristics of Akt phosphorylation in cells expressing YFP-EGFR-ICD Del746. On one hand, pAkt showed a preferential localization to membrane ruffles, and often accumulated at the tip of lamellipodial protrusions (arrowhead). On the other hand, cells expressing high (cell#1) or low (cell#2) levels of YFP-EGFR-ICD Del746, often contained similar levels of pAkt.

MCF-7 cells transfected with YFP-EGFR-ICD wt, Del746 or V948R, were immunostained using specific antibodies to detect endogenous phosphorylated Akt (pAkt-S473) and phosphorylated ERK (pERK-T202/Y204). The levels of pAkt (Figure 2A) and pERK (Figure 2B) were undetectable in non transfected cells as well as in cells expressing YFP-EGFR-ICD wt or YFP-EGFR-ICD V948R. In contrast, phosphorylation of both Akt and ERK was clearly detected by immunofluorescence in YFP-EGFR-ICD Del746-transfected cells. A more intense signal was consistently obtained with the anti-pAkt antibody, which was therefore used in subsequent experiments. As illustrated in Figure 2C, phosphorylated Akt in YFP-EGFR-ICD Del746-transfected MCF-7 cells localized preferentially to membrane ruffles and the tip of the lamellipodial protrusions mentioned above. Remarkably, we did not observe a correlation between the expression levels of ectopic EGFR ICD and the intensity of the endogenous pAkt signal.

### The sensitivity of EGFR mutants to erlotinib can be evaluated in the context of YFP-EGFR-ICD

**Figure 3:**
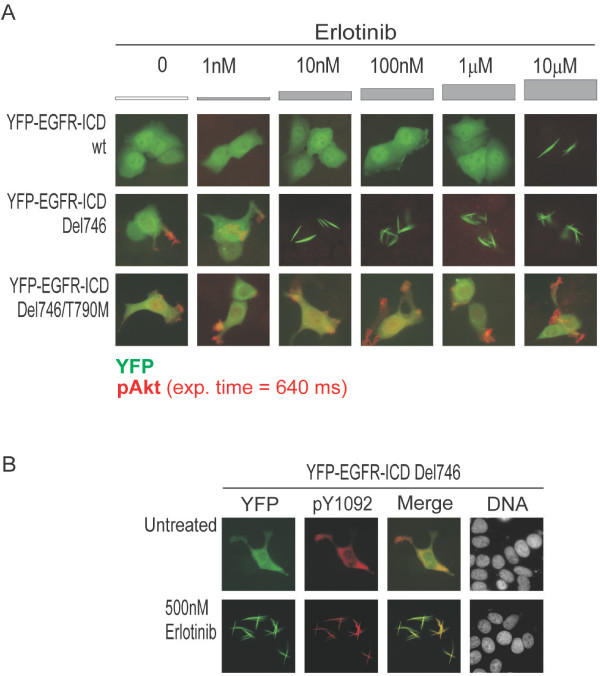
**Evaluating erlotinib sensitivity of EGFR mutants in the context of YFP-EGFR-ICD**. *A*. Representative examples of MCF-7 cells expressing YFP-EGFR-ICD wild type, Del746 or Del746/T790M (green), stained for endogenous phosphorylated Akt (red). Cells were treated for 20 hours with the indicated concentration of erlotinib. The different response of each EGFR variant to erlotinib treatment is readily visualized by immunoflourescence. YFP-EGFR-ICD wild type does not induce Akt phosphorylation, and relocates into thick cytoplasmic fibrils at 10 μM erlotinib. One thousand-fold lower concentration of the drug (10 nM) inhibited Del746-induced Akt phosphorylation, and caused fibrilar relocation of the ectopic protein. The double mutant Del746/T790M did not form fibrils and induced Akt phosphorylation even in the presence of 10 μM erlotinib. *B*. Images show that YFP-EGFR-ICD Del746 (green) remains phosphorylated at Y1092 (red) after relocating into fibrils in the presence of erlotinib.

We next evaluated the response of the Del746 mutant in the context of YFP-EGFR-ICD to the TKI inhibitor erlotinib. The TKI-resistant double mutant Del746/T790M [24,25] was also tested. Four hours post-transfection, erlotinib at a final concentration ranging from 1 nM to 10 μM was added to the culture medium, and cells were incubated for 20 hours. Samples were then fixed and immunostained using the anti-pAkt antibody. As illustrated in Figure 3A, no effect of erlotinib on cells expressing YFP-EGFR-ICD wt was noted up to 1 μM. At 10 μM, erlotinib induced the relocation of the chimeric protein to thick cytoplasmic filaments, reminiscent of actin cables [26]. EGFR can interact with actin [27], and the ICD fragment used in our assay includes the actin binding domain of EGFR. However, rhodamine-conjugated phalloidin failed to show co-localization with YFP-EGFR-ICD filaments (data not shown), suggesting that they do not contain actin. In cells expressing YFP-EGFR-ICD Del746, Akt phosphorylation was detected in both untreated samples and samples treated with 1 nM erlotinib. However 10 nM or higher concentration of the drug abrogated Akt phosphorylation and induced fibril formation. Surprisingly, Y1092 phosphorylation of YFP-EGFR-ICD Del746 was still detected in these fibrils (Figure 3C). In line with previous observations [25] the T790M mutation abrogated erlotinib sensitivity. Thus, Akt phosphorylation was readily detected in cells expressing YFP-EGFR-ICD Del746/T790M, even after treatment with 10 μM erlotinib.

### Testing the kinase activity and erlotinib sensitivity of uncommon EGFR mutants using the YFP-EGFR-ICD assay

**Figure 4:**
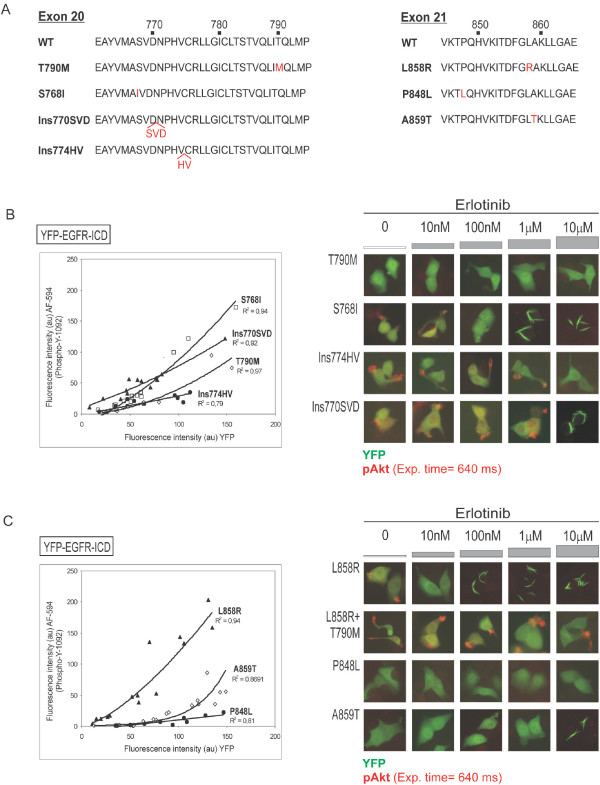
**Testing the kinase activity and erlotinib sensitivity of uncommon EGFR mutants using the YFP-EGFR-ICD assay**. *A*. Partial amino acid sequence of EGFR exon 20 and exon 21 illustrating the location of the mutations examined (red letters). *B*. Kinase activity and erlotinib sensitivity of different exon 20 mutations. Graph shows that autophosphorylation levels are lower for T790M (white diamonds) than for S768I (white squares) or Ins770SVD (black triangles). Low expression levels hampered the accurate evaluation of Ins774HV (black circles). Images show that YFP-EGFR T790M did not effectively induce phosphorylation of endogenous Akt in MCF-7 cells, and did not relocate into fibrils upon erlotinib treatment. S768I-induced pAkt was inhibited by 100 nM erlotinib and the ectopic protein relocated into fibrils at 1 μM. The phosphorylation of Akt induced by exon 20 insertions was only inhibited at 10 μM erlotinib. This drug concentration also induced relocation of YFP-EGFR-ICD Ins770SVD into fibrils. *C*. Kinase activity and erlotinib sensitivity of different exon 21 mutations. Graph shows that the common L858R mutation confers higher autophosphorylation levels to YFP-EGFR-ICD than P848L and A859T. Images show that, unlike L858R, these uncommon exon 21 mutants did not induce phosphorylation of endogenous Akt. Erlotinib blocked L858R-induced pAkt at 10 nM, and caused relocation of the ectopic protein into fibrils at 100 nM. Both effects were readily abrogated by the TKI-resistant mutation T790M. In all cases, data corresponding to one experiment are shown. Each EGFR mutant was tested at least twice with similar results.

We next applied the YFP-EGFR-ICD-based assay to test several uncommon EGFR mutations (Figure 4A) on which limited or no biochemical information is available. These included exon 20 insertions Ins770SVD and Ins774HV, and the exon 21 point mutation P848L, which were detected during our analysis of NSCLC samples (unpublished data). We also tested the exon 21 mutation A859T identified by other groups [28-30].

These mutations were introduced into YFP-EGFR-ICD and transient transfection experiments were carried out in MCF-7 cells. The kinase activity of each mutant (autophosphorylation at Y1092 and phosphorylation of endogenous Akt), and its response to different concentrations of erlotinib were evaluated as described above. Other exon 20 and 21 mutations (T790M, S768I and L858R) that have been previously tested using transfection-based assays were also included in the assay for comparison.

Among the exon 20 mutations tested, Ins770SVD showed an intermediate level of autophosphorylation, lower than S768I, but higher than T790M (Figure 4B). It was not possible to accurately evaluate autophosphorylation of Ins774HV, since the expression level of this mutant was consistently low in all attempted experiments. Akt phosphorylation was readily detected in cells transfected with S768I, Ins770SVD or Ins774HV, but not in cells expressing T790M (Figure 4B). The phosphorylation of Akt induced by S768I was abrogated by 100 nM erlotinib, and fibril formation was noted upon treatment with 1 μM or higher concentration of the drug. In contrast, the phosphorylation of Akt induced by Ins770SVD or Ins774HV was only abrogated at the highest concentration of erlotinib tested (10 μM). In the case of YFP-EGFR-ICD Ins770SVD, 10 μM erlotinib also induced relocation of the chimeric protein to fibrils. No fibrils were observed in the case of YFP-EGFR-ICD T790M or Ins774HV at any of the erlotinib concentrations tested.

On the other hand, YFP-EGFR-ICD proteins bearing the uncommon exon 21 mutation P848L and A859T showed markedly lower autophosphorylation levels than YFP-EGFR-ICD L858R (Figure 4C). Neither P848L nor A859T were able to induce phosphorylation of endogenous Akt. Erlotinib induced fibrilar relocation of YFP-EGFR-ICD A859T when applied at 10 μM, but did not have any apparent effect on P848L at any of the concentrations tested. In contrast, YFP-EGFR-ICD L858R induced phosphorylation of endogenous Akt, which was inhibited by 10 nM of erlotinib, and 100 nM or higher concentrations of the drug induced fibril formation. The T790M mutation abrogated the effect of erlotinib on L858R, and the L858R/T790M double mutant readily induced Akt phosphorylation even in the presence of 10 μM erlotinib.

## Discussion

In a subset of NSCLC patients, the presence of somatic mutations in the kinase domain of EGFR may predict the outcome of treatment with the EGFR TKIs erlotinib and gefitinib. The favorable clinical response of tumors harboring the common exon 19 deletions or the L858R mutation correlates with the high TKI sensitivity of these EGFR mutant proteins at the molecular level. However, a large variety of different EGFR mutant alleles have been identified in NSCLC patients, and it is becoming increasingly clear that different EGFR mutants may vary dramatically in their sensitivity or resistance to TKIs [15,31-33]. This issue is of particular importance in the context of on-going prospective clinical studies in which patient selection is based on the presence of EGFR mutations. In addition, novel treatment options are being explored for those NSCLC patients bearing TKI-resistant EGFR mutations. These options include the use of second-generation irreversible EGFR TKIs currently on development, but might be extended in the future to targeting other components of the pathway.

Evaluating the biochemical characteristics of mutant EGFR proteins using *in vitro *or cell-based assays provides clues to the phenotypical consequences of each alteration. Several relatively uncommon EGFR mutants have been tested in transfection-based assays, using immunoblot with phosphorylation-specific antibodies to assess EGFR activity [15,17-21]. In these studies, a homogeneous population of transfected cells was usually generated, by using viral transduction or by selecting stable transfectants, which are both labor-intensive and time-consuming procedures. We describe here a rapid cellular assay system to evaluate the kinase activity and erlotinib sensitivity of EGFR mutants, using an YFP-tagged fragment of EGFR intracellular domain (YFP-EGFR-ICD) and immunofluorescence. Our assay presents several advantageous characteristics with respect to previously used methods. First, by using a fragment of the receptor lacking the extracellular domain one would expect to reduce interference from the experimental context, which may have been partially responsible for some controversial findings [4,6,10,19,20]. Furthermore, the use of a shorter EGFR fragment instead of the full-length receptor renders the mutagenesis procedure more efficient. Finally, by evaluating EGFR activity in a single-cell basis, our assay circumvents the need of a homogeneous population of transfected cells allowing the use of transient transfection. The whole procedure of testing a new EGFR mutant, including site-directed mutagenesis (2 days), verification of the construct by sequencing (1 day), transfection, immunostaining and scoring (3 days) can be completed in approximately one week. In comparison, the generation of a population of stably transfected cells would typically require several weeks of selection in antibiotic-containing medium.

By using the well-characterized EGFR mutant Del746 as control, we demonstrate that the YFP-EGFR-ICD-based assay readily identifies differences between this mutant and the wild type protein in terms of autophosphorylation, activation of downstream signaling pathways and sensitivity to erlotinib. We also show that computer-assisted measurement of fluorescence intensity can be used to obtain a semi-quantitative comparison of autophosphorylation levels between different mutants. It must be acknowledged that immunofluorescence staining is a less quantitative approach than immunoblot to evaluate protein phosphorylation levels, a disadvantage that is, in our view, counterbalanced by the preservation of cellular morphology. Morphological examination allowed us to observe that phosphorylated endogenous Akt preferentially localizes to membrane ruffles and the tip of lamellipodial protrusions in cells expressing mutant EGFR-ICDs. This observation is consistent with the localization of activated endogenous Akt in growth factor-stimulated cells and its role in cell motility [[34], and references therein].

Microscopy analysis led to the unexpected observation that erlotinib treatment induces the relocation of the YFP-EGFR-ICD chimeric protein to thick cytoplasmic filaments. Importantly, the wild type protein formed fibrils only at 10 μM erlotinib, whereas the TKI-sensitive mutants relocated to fibrils in the presence of 10–100 nM erlotinib, and this effect was fully abrogated by the erlotinib-resistant T790M mutation. These observations suggest that the relocation of the chimeric YFP-EGFR-ICD protein into thick fibrils at lower drug concentrations is a marker of erlotinib sensitivity in our assay. The molecular basis for this effect of erlotinib is presently unclear. Additional experiments are required, for example, to clarify why YFP-EGFR-ICD Del746 remains phosphorylated (pY1092) in these fibrils in the presence of 500 nM erlotininb, even if downstream signaling (pAkt) is inhibited. We speculate that erlotinib binding to the ATP-binding site in the context of YFP-EGFR-ICD molecule may introduce a conformational alteration sufficient to lead to the aggregation of the chimeric protein. Importantly, we have noted that such effect is not erlotinib-specific, since a similar relocation can be induced by gefitinib treatment (data not shown). It remains to be further examined if fibril formation is a general effect of TKI-mediated EGFR inhibition. In this case, the YFP-EGFR-ICD assay system could be adapted for high content screening of potential anti-EGFR agents, since the shift from a diffuse YFP signal to a fluorescent signal concentrated in thick fibrils would be readily detected using automated image analysis. This possibility is particularly appealing in the context of the currently on-going effort to develop second-generation irreversible TKIs and other agents that may circumvent resistance to TKI associated wit the presence of certain types of EGFR mutations.

Over the last three years, we have carried out mutational analysis of EGFR in tumor samples from nearly 300 NSCLC patients [[35,36] and unpublished data]. Several uncommon EGFR mutants identified in the course of this analysis were tested using the YFP-EGFR-ICD assay.

Mutations in exon 20, most notably T790M, are usually associated with resistance to gefitinib and erlotinib [reviewed in [37]]. We found that Ins770SVD and Ins774HV are more resistant to erlotinib than S768I in our assay. These results are in line with previous data showing increased erlotinib resistance for a similar (Ins770NPG) mutant [15]. Furthermore, our results show that both insertions confer higher kinase activity than T790M, thus underscoring an important difference between these two types of exon 20 alterations. In this regard, there has been some controversy regarding the enhanced kinase activity of T790M-mutant EGFR [22]. We noted that the autophosphorylation level (pY1092) of YFP-EGFR-ICD T790M was indeed higher than that of the wild type protein at lower expression levels (compare graphs in Figure 1D and Figure 4B), as reported by Vikis et al [22]. However, the ability of this mutant to activate downstream signaling was clearly reduced in comparison to the ICD constructs bearing the Del746, L858R, S768I, Ins770SVD or Ins774HV mutations. Recent structural analyses indicate that enhanced activity of EGFR mutants may derive form the disruption of autoinhibitory interactions that suppress EGFR basal activity [23,38]. Our data suggest that exon 20 insertions may disrupt these interactions to a greater extent than the T790M point mutation.

On the other hand, our functional analysis indicates that the uncommon exon 21 mutation P848L is not a kinase-activating mutation and does not confer increased sensitivity to erlotinib. This change has been detected in both tumor and normal tissues from NSCLC patients [[39]; Gallegos-Ruiz et al., unpublished data]. Furthermore, although EGFR and K-ras mutations are in general mutually exclusive in NSCLC patients [37], a K-Ras mutation (G12V) was detected in tumor cells bearing the P848L allele (Gallegos-Ruiz et al., unpublished). Like P848L, the A859T variant, which has been detected in two non-responding NSCLC patients [28,29], did not confer increased kinase activity or erlotinib sensitivity in our test. Together, the results from the YFP-EGFR-ICD assay and the clinical behavior of tumors bearing these alterations suggest that P848L and A859T are likely to be uncommon, functionally silent EGFR polymorphisms.

## Conclusion

In conclusion, we describe here the use of a simple cellular assay that can be easily implemented to functionally evaluate EGFR variants. The ability to rapidly obtain functional information on EGFR variants of unknown relevance might prove important in the future for the management of NSCLC patients bearing uncommon EGFR mutations. In addition, our assay may be used to determine the response of resistant EGFR mutants to novel second-generation TKIs or to other therapeutic agents targeting the EGFR signaling pathway.

## Methods

### Plasmid construction and site-directed mutagenesis

In order to generate the YFP-EGFR-ICD construct, a DNA fragment encoding EGFR residues 688–1116 was amplified by PCR using primers TDG1 and TDG4, and full-length human EGFR cDNA (kindly provided by Dr. H. Nakagawa, University of Pennsylvania, Philadelphia) as template. The amplified product was digested with HindIII and KpnI and cloned into the pEYFP-C1 mammalian expression vector (Clontech, Palo Alto, CA). NSCLC- associated mutations were subsequently introduced into YFP-EGFR-ICD using the QuickChange II XL Site-Directed Mutagenesis Kit (Stratagene, La Jolla, CA) following manufacturer's protocol. In all cases, the sequence of the inserts was verified by DNA sequence. The sequence of all primers used is available upon request.

### Cell culture, transfection and drug treatment

Human breast cancer cells MCF-7 were grown in Dulbecco's modified Eagle's medium (BioWhittaker, Walkersville, MD), supplemented with 10% fetal calf serum (FCS), 100 units/ml penicillin, and 100 μg/ml streptomycin (Gibco-Invitrogen, Breda, The Netherlands). Cells were seeded onto sterile glass coverslips in twelve-well trays, and transfected with 0.5–1 μg of plasmid DNA using the FuGene6 transfection reagent (Roche Molecular Biochemicals, Almere, The Netherlands), following the manufacturer's protocol. Erlotinib (Roche Pharmaceuticals, Mannheim, Germany) was added at the indicated concentration 4 hours after transfection, and the cells were incubated for 20 hours before being processed for immunofluorescence analysis. Erlotinib treatment was always performed in standard culture medium containing 10% FCS.

### Immunofluorescence and microscopy analysis

To evaluate EGFR autophosphorylation, rabbit anti-pEGFR-Y845 (#2231, diluted 1:180) and mouse anti-pEGFR-Y1068 (#2236, diluted 1:180) antibodies were used. Note that the EGFR numbering system used by the manufacturer (Cell Signaling Technology, Danvers, MA) does not include the 24-residue signal peptide. According to the numbering system used in this report, these antibodies recognize residues pY869 and pY1092, respectively. On the other hand, rabbit anti-pAkt-S473 (#9271, diluted 1:100) and rabbit anti-pERK1/ERK2-T202/Y204 (#9101, diluted 1:100) antibodies, both from Cell Signaling Technology, were used to evaluate activation status of EGFR downstream pathways.

The immunostaining procedure was as previously described [40] with minor modifications. Briefly, cells were fixed using 3.7% formaldehyde in PBS for 30 minutes and permeabilized with 0.2% Triton X-100 in PBS for 10 minutes. Following a blocking step with 3% bovine serum albumin in PBS for 1 hour, the primary antibody diluted in blocking solution was applied for 1 hour. After washing with PBS, samples were incubated with Alexa Fluor 594 (AF-594)-conjugated anti-mouse or anti-rabbit secondary antibodies (Molecular Probes-Invitrogen, Breda, The Netherlands) for 45 min. Finally, the coverslips were mounted onto microscope slides with Vectashield (Vector, Burlingame, CA). The chromosome stain Hoechst 33285(Sigma, St Louis, MO) was used to counterstain the cell nuclei.

F-actin staining was carried out by incubating fixed and permeabilized cells with Rhodamine-conjugated phalloidin diluted in blocking solution for 30 minutes (Molecular Probes-Invitrogen, Breda, The Netherlands).

Slides were examined using an inverted Leica DMIRB/E fluorescence microscope (Leica Heidelberg, Heidelberg, Germany). The LeicaQ500MC Quantimet software V01.01 (Leica Cambridge Ltd., Cambridge, UK) was used to collect images, keeping exposure time constant to allow for comparison of signal intensity between different samples. The same software was used to carry out semi-quantitative image analysis of YFP-EGFR-ICD expression level and pY1092 phosphorylation level. To this end, images were acquired using 400 × magnification. A cytoplasmic area was selected (Figure 1D), and the intensity of the fluorescent signal within this region was measured in the green (YFP fluorophore) and the red (AF-594 fluorophore) channels. The intensity of the signal was also measured in a region outside the cell to determine background fluorescence. After subtracting the background, the fluorescence intensity of both fluorophores was plotted against each other using Excel, and the best-fitting trend line (highest R^2 ^value) was added using the "Add trend line" feature.

## Competing interests

J.A.R. received financial support from Roche during the elaboration of this study. G.G. received research grants from Roche and Astra Zeneca, and was a consultant for both companies.

## Authors' contributions

MM dG generated the plasmids and carried out cellular assays. MI G-R sequenced the plasmids and carried out cellular assays. GG participated in the interpretation of the results and the drafting of the manuscript. JAR conceived the study, carried out experimental work and participated in the interpretation of the results and in the drafting of the manuscript. All authors read and approved the final manuscript.
